# Supplementary material for: End Sequence Analysis Toolkit (ESAT) expands the extractable information from single-cell RNA-seq data
Source: Genome Res. 2016 Oct;26(10):1397–410. doi: 10.1101/gr.207902.116 (PMC5052061; doi:10.1101/gr.207902.116)
Supplement: Supplemental Material [file supp_gr.207902.116_Supp_Materials.tar.gz › ESAT-esat_v0.1/doc/html/classbroad_1_1core_1_1sequence_1_1_sequence_region.html]

ESAT: broad.core.sequence.SequenceRegion Class Reference


ESAT


- Main Page
- Related Pages
- Packages
- Classes
- Files

- Class List
- Class Index
- Class Hierarchy
- Class Members

All Classes Namespaces Files Functions Variables Enumerator Pages

- broad
- core
- sequence
- SequenceRegion

Public Member Functions |
Static Public Attributes |
List of all members

broad.core.sequence.SequenceRegion Class Reference

Inheritance diagram for broad.core.sequence.SequenceRegion:

[legend]

Collaboration diagram for broad.core.sequence.SequenceRegion:

[legend]

|  |  |
| --- | --- |
| Public Member Functions | |
|  | SequenceRegion (String containingSequenceId) |
|  | |
|  | SequenceRegion (String containingSequenceId, String chr, int start, int end) |
|  | |
|  | SequenceRegion (String containingSequenceId, Annotation annotation) |
|  | |
| int | getRegionEnd () |
|  | |
| void | setRegionEnd (int regionEnd) |
|  | |
| int | getRegionStart () |
|  | |
| void | setRegionStart (int regionStart) |
|  | |
| WindowSlider | getSlider (int windowSize, int overlap) |
|  | |
| int | length () |
|  | |
| Strand | getOrientation () |
|  | |
| void | setReversedOrientation (boolean reversed) |
|  | |
| String | getContainingSequenceId () |
|  | |
| String | getId () |
|  | |
| int | getStart () |
|  | |
| int | getEnd () |
|  | |
| double | getScore () |
|  | |
| void | setScore (double score) |
|  | |
| double | getExtraScore (int i) |
|  | |
| String | getName () |
|  | |
| void | setId (String id) |
|  | |
| void | setName (String name) |
|  | |
| boolean | inReversedOrientation () |
|  | |
| long | getMiddle () |
|  | |
| void | setStart (int start) |
|  | |
| void | setEnd (int end) |
|  | |
| Sequence | getSequence () |
|  | |
| void | setSequence (Sequence seq) |
|  | |
| String | getChromosome () |
|  | |
| void | setChromosome (String chr) |
|  | |
| boolean | overlaps (LightweightGenomicAnnotation other, int buffer) |
|  | |
| boolean | overlaps (LightweightGenomicAnnotation other) |
|  | |
| int | getOverlap (LightweightGenomicAnnotation other) |
|  | |
| Annotation | minus (Annotation other) |
|  | |
| Annotation | minus (List<?extends Annotation > others) |
|  | |
| void | takeIntersection (LightweightGenomicAnnotation other) |
|  | |
| void | takeUnion (LightweightGenomicAnnotation other) |
|  | |
| void | stitchTo (LightweightGenomicAnnotation other) |
|  | |
| boolean | isFlankedBy (TwoSubjectAnnotation twoSubjectAnnotation, int buffer) |
|  | |
| int | getFivePrimeBases () |
|  | |
| int | getThreePrimeBases () |
|  | |
| String | toString () |
|  | |
| int | getAbsoluteStart (int absoluteStart) |
|  | |
| int | getAbsoluteEnd (int absoluteStart) |
|  | |
| String | getLocationString () |
|  | |
| String | getChromosomeString () |
|  | |
| SequenceRegion | extractRegionBasedOnGC (float targetGC, int size, int buffer) |
|  | |
| boolean | contains (LightweightGenomicAnnotation other) |
|  | |
| int | getDistanceTo (LightweightGenomicAnnotation other) |
|  | |
| void | setOrientation (String orientation) |
|  | |
| int | compareTo (GenomicAnnotation arg0) |
|  | |
| List< Annotation > | disect (Annotation a) |
|  | |
| List< Annotation > | disect (List<?extends Annotation > disectors) |
|  | |
| SequenceRegion | getRegion (int start, int end) |
|  | |
| int | getOrientedEnd () |
|  | |
| int | getOrientedStart () |
|  | |
| boolean | isUnoriented () |
|  | |
| void | addBlock (String name, int start, int end) |
|  | |
| List<?extends Annotation > | getBlocks () |
|  | |
| List<?extends Annotation > | getBlocks (boolean oriented) |
|  | |
| boolean | mayHaveBlocks () |
|  | |
| String | toUCSC () |
|  | |
| int | getLength () |
|  | |
| boolean | overlaps (Collection<?extends Annotation > others, int buffer) |
|  | |
| boolean | overlaps (Collection<?extends Annotation > others) |
|  | |
| void | removeExtraScores () |
|  | |
| int | compareTo (LightweightGenomicAnnotation b) |
|  | |
| LightweightGenomicAnnotation | intersect (LightweightGenomicAnnotation other) |
|  | |
| List< Annotation > | intersect (List<?extends Annotation > annotations) |
|  | |
| double | percentGC () |
|  | |
| void | addExtraScore (double score) |
|  | |
| List< Double > | getExtraScores () |
|  | |
| boolean | almostEqual (LightweightGenomicAnnotation other, int fudge) |
|  | |
| int | getSAMStart () |
|  | |
| int | getSAMEnd () |
|  | |
| String | getReferenceName () |
|  | |
| String | getChr () |
|  | |
| Strand | getStrand () |
|  | |
| boolean | hasOrientation () |
|  | |
| int | numBlocks () |
|  | |
| int | getSize () |
|  | |
| int | size () |
|  | |
| int | getLengthOnReference () |
|  | |
| int | getReferenceCoordinateAtPosition (int positionInAnnotation) |
|  | |
| int | getPositionAtReferenceCoordinate (int referenceCoordinate) |
|  | |
| int | getReferenceCoordinateAtPosition (int positionInAnnotation, boolean ignoreOrientation) |
|  | |
| int | getPositionAtReferenceCoordinate (int referenceCoordinate, boolean ignoreOrientation) |
|  | |
| void | setOrientation (char orientation) |
|  | |
| void | setOrientation (Strand orientation) |
|  | |
| void | setOrientedStart (int orientedStart) |
|  | |
| void | setOrientedEnd (int orientedEnd) |
|  | |
| void | setReferenceName (String refName) |
|  | |
| boolean | equals (Annotation other) |
|  | |
| void | expand (int deltaStart, int deltaEnd) |
|  | |
| Annotation | trim (int deltaStart, int deltaEnd) |
|  | |
| Annotation | copy () |
|  | |
| int | getDistanceTo (Annotation other) |
|  | |
| String | toBED () |
|  | |
| String | toShortBED () |
|  | |
| String | toBEDGraph () |
|  | |
| boolean | overlaps (Annotation other, int buffer) |
|  | |
| boolean | overlaps (Annotation other) |
|  | |
| boolean | overlaps (Annotation other, int buffer, boolean considerStrand) |
|  | |
| boolean | overlapsStranded (Annotation other) |
|  | |
| int | getOverlap (Annotation other) |
|  | |
| boolean | contains (Annotation other) |
|  | |
| Annotation | union (Annotation other) |
|  | |
| Annotation | intersect (Annotation other) |
|  | |
| int | compareToAnnotation (Annotation b) |
|  | |
| void | stitchTo (Annotation next) |
|  | |
| String | toCigar () |
|  | |
| boolean | fullyContains (Annotation annotation) |
|  | |
| int | compareTo (Annotation arg0) |
|  | |
| void | moveToCoordinate (int i) |
|  | |
| void | shift (int delta) |
|  | |
| boolean | isNegativeStrand () |
|  | |
| Annotation | complement () |
|  | |
| Annotation | minus (Collection<?extends Annotation > others) |
|  | |
| boolean | equals (Annotation other, boolean useOrientation) |
|  | |
| Collection<?extends Annotation > | getSpliceConnections () |
|  | |
| String | toBED (int r, int g, int b) |
|  | |
| boolean | overlaps (Annotation other, boolean considerOrientation) |
|  | |
| int | getMidpoint () |
|  | |
| String | getFullInfoString () |
|  | |
| Public Member Functions inherited from broad.core.sequence.Sequence | |
|  | Sequence (String id) |
|  | |
|  | Sequence (String id, boolean isLarge) |
|  | |
|  | Sequence (String id, int expectedSize) |
|  | |
| String | getId () |
|  | |
| void | setForwardStrand (boolean isForwardStrand) |
|  | |
| void | setId (String id) |
|  | |
| void | unloadSequence () |
|  | |
| void | unloadEncodedSequence () |
|  | |
| void | unloadAllSequences () |
|  | |
| int | getLength () |
|  | |
| void | setCharAt (int position, char newCharacter) throws IllegalAccessException |
|  | |
| String | getSequenceBases () |
|  | |
| void | setSequenceBases (String sequence) |
|  | |
| void | append (String partialSequence) |
|  | |
| Sequence | shuffle () |
|  | |
| void | append (char c) |
|  | |
| boolean | isGap (int position) |
|  | |
| List< GenomicAnnotation > | getSoftmaskedRegions () |
|  | |
| int | countSoftMaskedBases () |
|  | |
| void | maskSoftmaskedRegions () |
|  | |
| short[] | getEncodedSequence () |
|  | |
| Matrix | getVectorEncodedSequence () |
|  | |
| Matrix | encodeSequenceAsVector () |
|  | |
| short[] | encodeSequence () |
|  | |
| short[] | encodeSequenceIgnoreCase () |
|  | |
| short[] | encodeSequenceIgnoreCase (boolean distinguishMissingSequence) |
|  | |
| List< Short > | compute454Flow (char[] flowOrder) |
|  | |
| List< Short > | compute454Flow (char[] flowOrder, int start, int end) |
|  | |
| float | gcContent () |
|  | |
| boolean | contains (String motif) |
|  | |
| void | reverse () |
|  | |
| void | uppercase () |
|  | |
| void | mask (Annotation region, boolean softmask) |
|  | |
| SequenceRegion | getRegion (int start, int end) |
|  | |
| void | getRegion (SequenceRegion region) |
|  | |
| void | getRegion (SequenceRegion region, boolean softmask) |
|  | |
| void | getRegion (SequenceRegion region, boolean softmask, Map< String, IntervalTree< Alignments >> okRepeats) |
|  | |
| void | getRegions (List<?extends SequenceRegion > regions) |
|  | |
| SequenceRegion | extractRegionBasedOnGC (float targetGC, int size, int buffer) |
|  | |
| boolean | isForwardStrand () |
|  | |
| StringBuilder | getSequenceBuilder () |
|  | |
| int | getGapsSize () |
|  | |
| WindowSlider | getSlider (int windowSize, int overlap) |
|  | |
| List< SequenceRegion > | chunk (int chunkSize, int chunkOverlap) |
|  | |
| int | getEnd () |
|  | |
| List< int[]> | findUngappedSequenceChunks () |
|  | |
| Collection< Annotation > | getMatches (String s, int windowStart, int windowEnd, boolean includeRC) |
|  | |
| Sequence | getSubSequence (SequenceRegion region, int extension) |
|  | |
| Sequence | getSubSequence (String name, int start, int end, int extension) |
|  | |
| Sequence | getSubSequence (String name, int start, int end) |
|  | |
| Sequence | getSubsequence (Gene annot) |
|  | |
| Sequence | getSubsequence (Annotation annot) |
|  | |
| void | setCapacity (int size) |
|  | |
| Sequence | scramble () |
|  | |
| Sequence | getAntisense () |
|  | |
| String | getRefseqGeneName () |
|  | |
| String | getRefseqSpeciesName () |
|  | |
| String | getRefseqRnaClassName () |
|  | |
| Map< String, KmerInfo > | buildKmerMap (int kmer) |
|  | |

|  |  |
| --- | --- |
| Static Public Attributes | |
| static final int | INF = Integer.MAX\_VALUE |
|  | |
| Static Public Attributes inherited from broad.core.sequence.Sequence | |
| static final char[] | SHORT\_READ\_FLOW = {'T','A','C','G'} |
|  | |
| static final char[] | LONG\_READ\_FLOW = {'G','A','T','C'} |
|  | |
| static final short | SHORT\_ENCODED\_A = 0 |
|  | |
| static final short | SHORT\_ENCODED\_C = 1 |
|  | |
| static final short | SHORT\_ENCODED\_G = 2 |
|  | |
| static final short | SHORT\_ENCODED\_T = 3 |
|  | |
| static final short | SHORT\_ENCODED\_GAP = 4 |
|  | |
| static final short | SHORT\_ENCODED\_a = 5 |
|  | |
| static final short | SHORT\_ENCODED\_c = 6 |
|  | |
| static final short | SHORT\_ENCODED\_g = 7 |
|  | |
| static final short | SHORT\_ENCODED\_t = 8 |
|  | |
| static final short | SHORT\_ENCODED\_N = 9 |
|  | |

|  |  |
| --- | --- |
| Additional Inherited Members | |
| Static Public Member Functions inherited from broad.core.sequence.Sequence | |
| static short[] | encodeSequence (StringBuilder bases) |
|  | |
| static short[] | encodeSequenceIgnoreCase (StringBuilder sequence) |
|  | |
| static short[] | encodeSequenceIgnoreCase (StringBuilder sequence, boolean distinguishMissingSequence) |
|  | |
| static float | computeGCContent (String dnaString) |
|  | |
| static float[] | computeSequenceComposition (String dnaString) |
|  | |
| static Sequence | reverseSequence (Sequence seq) |
|  | |
| static String | reverseSequence (String seq) |
|  | |
| static String | decode (short[] shortEncodedSequence) |
|  | |
| static String | generateRandomSequence (int oligoSize) |
|  | |
| static String | get3Prime (String seq, int numBases) |
|  | |
| static Collection< String > | generateAll5Mers () |
|  | |
| static Collection< String > | generateAllKmers (int k) |
|  | |
| static String | complement (String seq) |
|  | |

## Constructor & Destructor Documentation

|  |  |  |  |  |  |
| --- | --- | --- | --- | --- | --- |
| broad.core.sequence.SequenceRegion.SequenceRegion | ( | String | *containingSequenceId* | ) |  |

|  |  |  |  |
| --- | --- | --- | --- |
| broad.core.sequence.SequenceRegion.SequenceRegion | ( | String | *containingSequenceId*, |
|  |  | String | *chr*, |
|  |  | int | *start*, |
|  |  | int | *end* |
|  | ) |  |  |

|  |  |  |  |
| --- | --- | --- | --- |
| broad.core.sequence.SequenceRegion.SequenceRegion | ( | String | *containingSequenceId*, |
|  |  | Annotation | *annotation* |
|  | ) |  |  |

## Member Function Documentation

|  |  |  |  |
| --- | --- | --- | --- |
| void broad.core.sequence.SequenceRegion.addBlock | ( | String | *name*, |
|  |  | int | *start*, |
|  |  | int | *end* |
|  | ) |  |  |

|  |  |  |  |  |  |
| --- | --- | --- | --- | --- | --- |
| void broad.core.sequence.SequenceRegion.addExtraScore | ( | double | *score* | ) |  |

|  |  |  |  |
| --- | --- | --- | --- |
| boolean broad.core.sequence.SequenceRegion.almostEqual | ( | LightweightGenomicAnnotation | *other*, |
|  |  | int | *fudge* |
|  | ) |  |  |

Checks whether two annotations differ by a small (fudge) factor

Parameters
:   |  |  |
    | --- | --- |
    | fudge | Maximum difference at either end or start to consider similar |

Returns

|  |  |  |  |  |  |
| --- | --- | --- | --- | --- | --- |
| int broad.core.sequence.SequenceRegion.compareTo | ( | GenomicAnnotation | *arg0* | ) |  |

|  |  |  |  |  |  |
| --- | --- | --- | --- | --- | --- |
| int broad.core.sequence.SequenceRegion.compareTo | ( | LightweightGenomicAnnotation | *b* | ) |  |

|  |  |  |  |  |  |
| --- | --- | --- | --- | --- | --- |
| int broad.core.sequence.SequenceRegion.compareTo | ( | Annotation | *arg0* | ) |  |

|  |  |  |  |  |  |
| --- | --- | --- | --- | --- | --- |
| int broad.core.sequence.SequenceRegion.compareToAnnotation | ( | Annotation | *b* | ) |  |

|  |  |  |  |  |
| --- | --- | --- | --- | --- |
| Annotation broad.core.sequence.SequenceRegion.complement | ( |  | ) |  |

|  |  |  |  |  |  |
| --- | --- | --- | --- | --- | --- |
| boolean broad.core.sequence.SequenceRegion.contains | ( | LightweightGenomicAnnotation | *other* | ) |  |

|  |  |  |  |  |  |
| --- | --- | --- | --- | --- | --- |
| boolean broad.core.sequence.SequenceRegion.contains | ( | Annotation | *other* | ) |  |

|  |  |  |  |  |
| --- | --- | --- | --- | --- |
| Annotation broad.core.sequence.SequenceRegion.copy | ( |  | ) |  |

|  |  |  |  |  |  |
| --- | --- | --- | --- | --- | --- |
| List<Annotation> broad.core.sequence.SequenceRegion.disect | ( | Annotation | *a* | ) |  |

|  |  |  |  |  |  |
| --- | --- | --- | --- | --- | --- |
| List<Annotation> broad.core.sequence.SequenceRegion.disect | ( | List<?extends Annotation > | *disectors* | ) |  |

|  |  |  |  |  |  |
| --- | --- | --- | --- | --- | --- |
| boolean broad.core.sequence.SequenceRegion.equals | ( | Annotation | *other* | ) |  |

|  |  |  |  |
| --- | --- | --- | --- |
| boolean broad.core.sequence.SequenceRegion.equals | ( | Annotation | *other*, |
|  |  | boolean | *useOrientation* |
|  | ) |  |  |

|  |  |  |  |
| --- | --- | --- | --- |
| void broad.core.sequence.SequenceRegion.expand | ( | int | *deltaStart*, |
|  |  | int | *deltaEnd* |
|  | ) |  |  |

|  |  |  |  |
| --- | --- | --- | --- |
| SequenceRegion broad.core.sequence.SequenceRegion.extractRegionBasedOnGC | ( | float | *targetGC*, |
|  |  | int | *size*, |
|  |  | int | *buffer* |
|  | ) |  |  |

Here is the call graph for this function:

|  |  |  |  |  |  |
| --- | --- | --- | --- | --- | --- |
| boolean broad.core.sequence.SequenceRegion.fullyContains | ( | Annotation | *annotation* | ) |  |

|  |  |  |  |  |  |
| --- | --- | --- | --- | --- | --- |
| int broad.core.sequence.SequenceRegion.getAbsoluteEnd | ( | int | *absoluteStart* | ) |  |

Here is the call graph for this function:

|  |  |  |  |  |  |
| --- | --- | --- | --- | --- | --- |
| int broad.core.sequence.SequenceRegion.getAbsoluteStart | ( | int | *absoluteStart* | ) |  |

Here is the call graph for this function:

|  |  |  |  |  |
| --- | --- | --- | --- | --- |
| List<? extends Annotation> broad.core.sequence.SequenceRegion.getBlocks | ( |  | ) |  |

|  |  |  |  |  |  |
| --- | --- | --- | --- | --- | --- |
| List<? extends Annotation> broad.core.sequence.SequenceRegion.getBlocks | ( | boolean | *oriented* | ) |  |

|  |  |  |  |  |
| --- | --- | --- | --- | --- |
| String broad.core.sequence.SequenceRegion.getChr | ( |  | ) |  |

|  |  |  |  |  |
| --- | --- | --- | --- | --- |
| String broad.core.sequence.SequenceRegion.getChromosome | ( |  | ) |  |

Here is the caller graph for this function:

|  |  |  |  |  |
| --- | --- | --- | --- | --- |
| String broad.core.sequence.SequenceRegion.getChromosomeString | ( |  | ) |  |

|  |  |  |  |  |
| --- | --- | --- | --- | --- |
| String broad.core.sequence.SequenceRegion.getContainingSequenceId | ( |  | ) |  |

Here is the caller graph for this function:

|  |  |  |  |  |  |
| --- | --- | --- | --- | --- | --- |
| int broad.core.sequence.SequenceRegion.getDistanceTo | ( | LightweightGenomicAnnotation | *other* | ) |  |

|  |  |  |  |  |  |
| --- | --- | --- | --- | --- | --- |
| int broad.core.sequence.SequenceRegion.getDistanceTo | ( | Annotation | *other* | ) |  |

|  |  |  |  |  |
| --- | --- | --- | --- | --- |
| int broad.core.sequence.SequenceRegion.getEnd | ( |  | ) |  |

Here is the caller graph for this function:

|  |  |  |  |  |  |
| --- | --- | --- | --- | --- | --- |
| double broad.core.sequence.SequenceRegion.getExtraScore | ( | int | *i* | ) |  |

|  |  |  |  |  |
| --- | --- | --- | --- | --- |
| List<Double> broad.core.sequence.SequenceRegion.getExtraScores | ( |  | ) |  |

|  |  |  |  |  |
| --- | --- | --- | --- | --- |
| int broad.core.sequence.SequenceRegion.getFivePrimeBases | ( |  | ) |  |

|  |  |  |  |  |
| --- | --- | --- | --- | --- |
| String broad.core.sequence.SequenceRegion.getFullInfoString | ( |  | ) |  |

|  |  |  |  |  |
| --- | --- | --- | --- | --- |
| String broad.core.sequence.SequenceRegion.getId | ( |  | ) |  |

Here is the call graph for this function:

Here is the caller graph for this function:

|  |  |  |  |  |
| --- | --- | --- | --- | --- |
| int broad.core.sequence.SequenceRegion.getLength | ( |  | ) |  |

Here is the call graph for this function:

Here is the caller graph for this function:

|  |  |  |  |  |
| --- | --- | --- | --- | --- |
| int broad.core.sequence.SequenceRegion.getLengthOnReference | ( |  | ) |  |

|  |  |  |  |  |
| --- | --- | --- | --- | --- |
| String broad.core.sequence.SequenceRegion.getLocationString | ( |  | ) |  |

|  |  |  |  |  |
| --- | --- | --- | --- | --- |
| long broad.core.sequence.SequenceRegion.getMiddle | ( |  | ) |  |

|  |  |  |  |  |
| --- | --- | --- | --- | --- |
| int broad.core.sequence.SequenceRegion.getMidpoint | ( |  | ) |  |

Here is the call graph for this function:

|  |  |  |  |  |
| --- | --- | --- | --- | --- |
| String broad.core.sequence.SequenceRegion.getName | ( |  | ) |  |

Here is the call graph for this function:

Here is the caller graph for this function:

|  |  |  |  |  |
| --- | --- | --- | --- | --- |
| Strand broad.core.sequence.SequenceRegion.getOrientation | ( |  | ) |  |

|  |  |  |  |  |
| --- | --- | --- | --- | --- |
| int broad.core.sequence.SequenceRegion.getOrientedEnd | ( |  | ) |  |

|  |  |  |  |  |
| --- | --- | --- | --- | --- |
| int broad.core.sequence.SequenceRegion.getOrientedStart | ( |  | ) |  |

|  |  |  |  |  |  |
| --- | --- | --- | --- | --- | --- |
| int broad.core.sequence.SequenceRegion.getOverlap | ( | LightweightGenomicAnnotation | *other* | ) |  |

|  |  |  |  |  |  |
| --- | --- | --- | --- | --- | --- |
| int broad.core.sequence.SequenceRegion.getOverlap | ( | Annotation | *other* | ) |  |

|  |  |  |  |  |  |
| --- | --- | --- | --- | --- | --- |
| int broad.core.sequence.SequenceRegion.getPositionAtReferenceCoordinate | ( | int | *referenceCoordinate* | ) |  |

|  |  |  |  |
| --- | --- | --- | --- |
| int broad.core.sequence.SequenceRegion.getPositionAtReferenceCoordinate | ( | int | *referenceCoordinate*, |
|  |  | boolean | *ignoreOrientation* |
|  | ) |  |  |

|  |  |  |  |  |  |
| --- | --- | --- | --- | --- | --- |
| int broad.core.sequence.SequenceRegion.getReferenceCoordinateAtPosition | ( | int | *positionInAnnotation* | ) |  |

Here is the caller graph for this function:

|  |  |  |  |
| --- | --- | --- | --- |
| int broad.core.sequence.SequenceRegion.getReferenceCoordinateAtPosition | ( | int | *positionInAnnotation*, |
|  |  | boolean | *ignoreOrientation* |
|  | ) |  |  |

|  |  |  |  |  |
| --- | --- | --- | --- | --- |
| String broad.core.sequence.SequenceRegion.getReferenceName | ( |  | ) |  |

|  |  |  |  |
| --- | --- | --- | --- |
| SequenceRegion broad.core.sequence.SequenceRegion.getRegion | ( | int | *start*, |
|  |  | int | *end* |
|  | ) |  |  |

Here is the call graph for this function:

|  |  |  |  |  |
| --- | --- | --- | --- | --- |
| int broad.core.sequence.SequenceRegion.getRegionEnd | ( |  | ) |  |

Here is the call graph for this function:

Here is the caller graph for this function:

|  |  |  |  |  |
| --- | --- | --- | --- | --- |
| int broad.core.sequence.SequenceRegion.getRegionStart | ( |  | ) |  |

Here is the caller graph for this function:

|  |  |  |  |  |
| --- | --- | --- | --- | --- |
| int broad.core.sequence.SequenceRegion.getSAMEnd | ( |  | ) |  |

|  |  |  |  |  |
| --- | --- | --- | --- | --- |
| int broad.core.sequence.SequenceRegion.getSAMStart | ( |  | ) |  |

|  |  |  |  |  |
| --- | --- | --- | --- | --- |
| double broad.core.sequence.SequenceRegion.getScore | ( |  | ) |  |

|  |  |  |  |  |
| --- | --- | --- | --- | --- |
| Sequence broad.core.sequence.SequenceRegion.getSequence | ( |  | ) |  |

|  |  |  |  |  |
| --- | --- | --- | --- | --- |
| int broad.core.sequence.SequenceRegion.getSize | ( |  | ) |  |

|  |  |  |  |
| --- | --- | --- | --- |
| WindowSlider broad.core.sequence.SequenceRegion.getSlider | ( | int | *windowSize*, |
|  |  | int | *overlap* |
|  | ) |  |  |

|  |  |  |  |  |
| --- | --- | --- | --- | --- |
| Collection<? extends Annotation> broad.core.sequence.SequenceRegion.getSpliceConnections | ( |  | ) |  |

|  |  |  |  |  |
| --- | --- | --- | --- | --- |
| int broad.core.sequence.SequenceRegion.getStart | ( |  | ) |  |

Here is the caller graph for this function:

|  |  |  |  |  |
| --- | --- | --- | --- | --- |
| Strand broad.core.sequence.SequenceRegion.getStrand | ( |  | ) |  |

|  |  |  |  |  |
| --- | --- | --- | --- | --- |
| int broad.core.sequence.SequenceRegion.getThreePrimeBases | ( |  | ) |  |

|  |  |  |  |  |
| --- | --- | --- | --- | --- |
| boolean broad.core.sequence.SequenceRegion.hasOrientation | ( |  | ) |  |

|  |  |  |  |  |
| --- | --- | --- | --- | --- |
| boolean broad.core.sequence.SequenceRegion.inReversedOrientation | ( |  | ) |  |

Here is the caller graph for this function:

|  |  |  |  |  |  |
| --- | --- | --- | --- | --- | --- |
| LightweightGenomicAnnotation broad.core.sequence.SequenceRegion.intersect | ( | LightweightGenomicAnnotation | *other* | ) |  |

|  |  |  |  |  |  |
| --- | --- | --- | --- | --- | --- |
| List<Annotation> broad.core.sequence.SequenceRegion.intersect | ( | List<?extends Annotation > | *annotations* | ) |  |

|  |  |  |  |  |  |
| --- | --- | --- | --- | --- | --- |
| Annotation broad.core.sequence.SequenceRegion.intersect | ( | Annotation | *other* | ) |  |

|  |  |  |  |
| --- | --- | --- | --- |
| boolean broad.core.sequence.SequenceRegion.isFlankedBy | ( | TwoSubjectAnnotation | *twoSubjectAnnotation*, |
|  |  | int | *buffer* |
|  | ) |  |  |

|  |  |  |  |  |
| --- | --- | --- | --- | --- |
| boolean broad.core.sequence.SequenceRegion.isNegativeStrand | ( |  | ) |  |

|  |  |  |  |  |
| --- | --- | --- | --- | --- |
| boolean broad.core.sequence.SequenceRegion.isUnoriented | ( |  | ) |  |

|  |  |  |  |  |
| --- | --- | --- | --- | --- |
| int broad.core.sequence.SequenceRegion.length | ( |  | ) |  |

Here is the call graph for this function:

Here is the caller graph for this function:

|  |  |  |  |  |
| --- | --- | --- | --- | --- |
| boolean broad.core.sequence.SequenceRegion.mayHaveBlocks | ( |  | ) |  |

|  |  |  |  |  |  |
| --- | --- | --- | --- | --- | --- |
| Annotation broad.core.sequence.SequenceRegion.minus | ( | Annotation | *other* | ) |  |

|  |  |  |  |  |  |
| --- | --- | --- | --- | --- | --- |
| Annotation broad.core.sequence.SequenceRegion.minus | ( | List<?extends Annotation > | *others* | ) |  |

|  |  |  |  |  |  |
| --- | --- | --- | --- | --- | --- |
| Annotation broad.core.sequence.SequenceRegion.minus | ( | Collection<?extends Annotation > | *others* | ) |  |

|  |  |  |  |  |  |
| --- | --- | --- | --- | --- | --- |
| void broad.core.sequence.SequenceRegion.moveToCoordinate | ( | int | *i* | ) |  |

|  |  |  |  |  |
| --- | --- | --- | --- | --- |
| int broad.core.sequence.SequenceRegion.numBlocks | ( |  | ) |  |

|  |  |  |  |
| --- | --- | --- | --- |
| boolean broad.core.sequence.SequenceRegion.overlaps | ( | LightweightGenomicAnnotation | *other*, |
|  |  | int | *buffer* |
|  | ) |  |  |

|  |  |  |  |  |  |
| --- | --- | --- | --- | --- | --- |
| boolean broad.core.sequence.SequenceRegion.overlaps | ( | LightweightGenomicAnnotation | *other* | ) |  |

|  |  |  |  |
| --- | --- | --- | --- |
| boolean broad.core.sequence.SequenceRegion.overlaps | ( | Collection<?extends Annotation > | *others*, |
|  |  | int | *buffer* |
|  | ) |  |  |

|  |  |  |  |  |  |
| --- | --- | --- | --- | --- | --- |
| boolean broad.core.sequence.SequenceRegion.overlaps | ( | Collection<?extends Annotation > | *others* | ) |  |

|  |  |  |  |
| --- | --- | --- | --- |
| boolean broad.core.sequence.SequenceRegion.overlaps | ( | Annotation | *other*, |
|  |  | int | *buffer* |
|  | ) |  |  |

|  |  |  |  |  |  |
| --- | --- | --- | --- | --- | --- |
| boolean broad.core.sequence.SequenceRegion.overlaps | ( | Annotation | *other* | ) |  |

|  |  |  |  |
| --- | --- | --- | --- |
| boolean broad.core.sequence.SequenceRegion.overlaps | ( | Annotation | *other*, |
|  |  | int | *buffer*, |
|  |  | boolean | *considerStrand* |
|  | ) |  |  |

|  |  |  |  |
| --- | --- | --- | --- |
| boolean broad.core.sequence.SequenceRegion.overlaps | ( | Annotation | *other*, |
|  |  | boolean | *considerOrientation* |
|  | ) |  |  |

|  |  |  |  |  |  |
| --- | --- | --- | --- | --- | --- |
| boolean broad.core.sequence.SequenceRegion.overlapsStranded | ( | Annotation | *other* | ) |  |

|  |  |  |  |  |
| --- | --- | --- | --- | --- |
| double broad.core.sequence.SequenceRegion.percentGC | ( |  | ) |  |

|  |  |  |  |  |
| --- | --- | --- | --- | --- |
| void broad.core.sequence.SequenceRegion.removeExtraScores | ( |  | ) |  |

|  |  |  |  |  |  |
| --- | --- | --- | --- | --- | --- |
| void broad.core.sequence.SequenceRegion.setChromosome | ( | String | *chr* | ) |  |

|  |  |  |  |  |  |
| --- | --- | --- | --- | --- | --- |
| void broad.core.sequence.SequenceRegion.setEnd | ( | int | *end* | ) |  |

Here is the call graph for this function:

|  |  |  |  |  |  |
| --- | --- | --- | --- | --- | --- |
| void broad.core.sequence.SequenceRegion.setId | ( | String | *id* | ) |  |

Here is the caller graph for this function:

|  |  |  |  |  |  |
| --- | --- | --- | --- | --- | --- |
| void broad.core.sequence.SequenceRegion.setName | ( | String | *name* | ) |  |

Here is the call graph for this function:

|  |  |  |  |  |  |
| --- | --- | --- | --- | --- | --- |
| void broad.core.sequence.SequenceRegion.setOrientation | ( | String | *orientation* | ) |  |

|  |  |  |  |  |  |
| --- | --- | --- | --- | --- | --- |
| void broad.core.sequence.SequenceRegion.setOrientation | ( | char | *orientation* | ) |  |

|  |  |  |  |  |  |
| --- | --- | --- | --- | --- | --- |
| void broad.core.sequence.SequenceRegion.setOrientation | ( | Strand | *orientation* | ) |  |

|  |  |  |  |  |  |
| --- | --- | --- | --- | --- | --- |
| void broad.core.sequence.SequenceRegion.setOrientedEnd | ( | int | *orientedEnd* | ) |  |

|  |  |  |  |  |  |
| --- | --- | --- | --- | --- | --- |
| void broad.core.sequence.SequenceRegion.setOrientedStart | ( | int | *orientedStart* | ) |  |

|  |  |  |  |  |  |
| --- | --- | --- | --- | --- | --- |
| void broad.core.sequence.SequenceRegion.setReferenceName | ( | String | *refName* | ) |  |

|  |  |  |  |  |  |
| --- | --- | --- | --- | --- | --- |
| void broad.core.sequence.SequenceRegion.setRegionEnd | ( | int | *regionEnd* | ) |  |

Here is the caller graph for this function:

|  |  |  |  |  |  |
| --- | --- | --- | --- | --- | --- |
| void broad.core.sequence.SequenceRegion.setRegionStart | ( | int | *regionStart* | ) |  |

Here is the caller graph for this function:

|  |  |  |  |  |  |
| --- | --- | --- | --- | --- | --- |
| void broad.core.sequence.SequenceRegion.setReversedOrientation | ( | boolean | *reversed* | ) |  |

|  |  |  |  |  |  |
| --- | --- | --- | --- | --- | --- |
| void broad.core.sequence.SequenceRegion.setScore | ( | double | *score* | ) |  |

|  |  |  |  |  |  |
| --- | --- | --- | --- | --- | --- |
| void broad.core.sequence.SequenceRegion.setSequence | ( | Sequence | *seq* | ) |  |

|  |  |  |  |  |  |
| --- | --- | --- | --- | --- | --- |
| void broad.core.sequence.SequenceRegion.setStart | ( | int | *start* | ) |  |

Here is the call graph for this function:

|  |  |  |  |  |  |
| --- | --- | --- | --- | --- | --- |
| void broad.core.sequence.SequenceRegion.shift | ( | int | *delta* | ) |  |

|  |  |  |  |  |
| --- | --- | --- | --- | --- |
| int broad.core.sequence.SequenceRegion.size | ( |  | ) |  |

Here is the caller graph for this function:

|  |  |  |  |  |  |
| --- | --- | --- | --- | --- | --- |
| void broad.core.sequence.SequenceRegion.stitchTo | ( | LightweightGenomicAnnotation | *other* | ) |  |

|  |  |  |  |  |  |
| --- | --- | --- | --- | --- | --- |
| void broad.core.sequence.SequenceRegion.stitchTo | ( | Annotation | *next* | ) |  |

|  |  |  |  |  |  |
| --- | --- | --- | --- | --- | --- |
| void broad.core.sequence.SequenceRegion.takeIntersection | ( | LightweightGenomicAnnotation | *other* | ) |  |

|  |  |  |  |  |  |
| --- | --- | --- | --- | --- | --- |
| void broad.core.sequence.SequenceRegion.takeUnion | ( | LightweightGenomicAnnotation | *other* | ) |  |

|  |  |  |  |  |
| --- | --- | --- | --- | --- |
| String broad.core.sequence.SequenceRegion.toBED | ( |  | ) |  |

|  |  |  |  |
| --- | --- | --- | --- |
| String broad.core.sequence.SequenceRegion.toBED | ( | int | *r*, |
|  |  | int | *g*, |
|  |  | int | *b* |
|  | ) |  |  |

|  |  |  |  |  |
| --- | --- | --- | --- | --- |
| String broad.core.sequence.SequenceRegion.toBEDGraph | ( |  | ) |  |

|  |  |  |  |  |
| --- | --- | --- | --- | --- |
| String broad.core.sequence.SequenceRegion.toCigar | ( |  | ) |  |

|  |  |  |  |  |
| --- | --- | --- | --- | --- |
| String broad.core.sequence.SequenceRegion.toShortBED | ( |  | ) |  |

|  |  |  |  |  |
| --- | --- | --- | --- | --- |
| String broad.core.sequence.SequenceRegion.toString | ( |  | ) |  |

Here is the call graph for this function:

|  |  |  |  |  |
| --- | --- | --- | --- | --- |
| String broad.core.sequence.SequenceRegion.toUCSC | ( |  | ) |  |

|  |  |  |  |
| --- | --- | --- | --- |
| Annotation broad.core.sequence.SequenceRegion.trim | ( | int | *deltaStart*, |
|  |  | int | *deltaEnd* |
|  | ) |  |  |

|  |  |  |  |  |  |
| --- | --- | --- | --- | --- | --- |
| Annotation broad.core.sequence.SequenceRegion.union | ( | Annotation | *other* | ) |  |

## Member Data Documentation

final int broad.core.sequence.SequenceRegion.INF = Integer.MAX\_VALUE

static

---

The documentation for this class was generated from the following file:

- java/broad/core/sequence/SequenceRegion.java


---

Generated on Fri Aug 15 2014 15:10:56 for ESAT by  

 1.8.7
